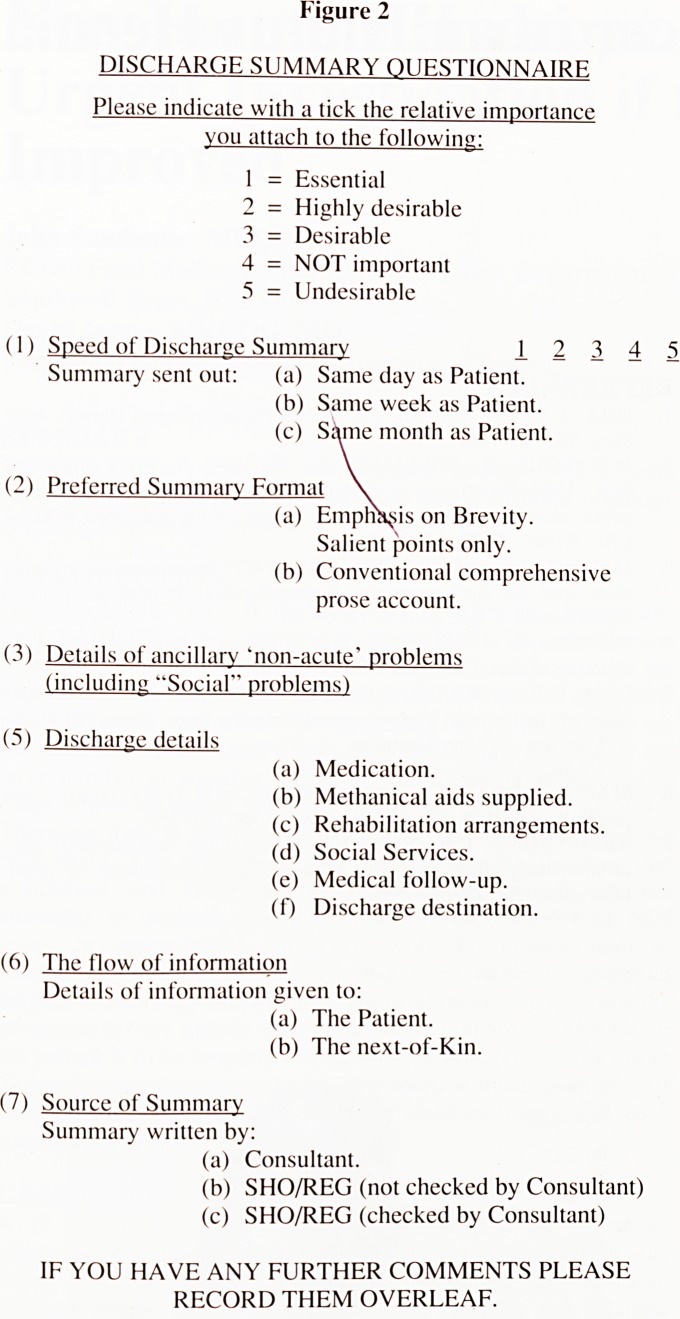# Towards Better Discharge Summaries

**Published:** 1991-06

**Authors:** M. H. King, S G Barber

**Affiliations:** General Practitioner N. Devon Health Care Trust; Physician in Care of the Elderly, N. Devon Health Care Trust

## Abstract

In an investigation of the communication between Hospital and General Practitioners, 99 General Practitioners were asked by means of a postal questionnaire to state the relative importance they attached to the issues of speed of delivery, format, author, and the content of the discharge summaries.

The issue of speed of delivery proved to be a central and recurrent theme in the replies received, with a clear demand for increased speed and efficiency in Hospital-General Practitioner communication.

In addition, an overwhelming support was revealed for summaries in the form of short prioritised problem lists, as opposed to longer conventional prose accounts. This response proved to be independent of the style of summary being received by the General Practitioners in the study.

With a clear need nationally to improve communication with General Practitioners, consideration should be given to adopting prioritised problem lists as a means of upgrading the quality of data sent to General Practitioners.


### Towards Better Discharge Summaries: Brevity and Structure

**M.H. King BM, DPD,** General Practitioner
**S G Barber BSc, MB, ChB, MRCP, DTM and H.,** Physician in Care of the Elderly, N. Devon Health Care Trust

### SUMMARY

In an investigation of the communication between Hospital and General Practitioners, 99 General Practitioners were asked by means of a postal questionnaire to state the relative importance they attached to the issues of speed of delivery, format, author, and the content of the discharge summaries.

The issue of speed of delivery proved to be a central and recurrent theme in the replies received, with a clear demand for increased speed and efficiency in Hospital-General Practitioner communication.

In addition, an overwhelming support was revealed for summaries in the form of short prioritised problem lists, as opposed to longer conventional prose accounts. This response proved to be independent of the style of summary being received by the General Practitioners in the study.

With a clear need nationally to improve communication with General Practitioners, consideration should be given to adopting prioritised problem lists as a means of upgrading the quality of data sent to General Practitioners.

### INTRODUCTION

Better communication within the health service has aroused increasing interest recently, with discharge summary reports proving to be an area of active controversy.

Renewed interest has led to a series of recent research initiatives, which have highlighted some serious shortcomings. An illustrative study completed in 1987 demonstrated that General Practitioners felt that "a delay, or lack of detail", materially affected their management in the cases of 24% of newly discharged patients[1]. A related study conducted in 1986 showed that over 50% of patients had contacted their General Practitioner before any information had been received from the hospital[2] This study also indicated that no information was received in an average of 11% of cases.

Several other studies have concentrated on the style or format of the summary. Ekeland & Castleden investigated the introduction of a shorter structured letter with interesting results, finding that 90% of General Practitioners considered the new format to be an inprovement, while 30% felt they arrived earlier, and 70% thought they were easier to read[3].

A third research area of interest has focussed on the content of the discharge letter. Previous evidence has confirmed that "Social topics" are perceived as valuable by General Practitioners, but are generally poorly covered in contemporary summaries, this being an area of consistent incongruity of emphasis between Hospital and General practitioners[4].

Against this background, and one of increasing pressure from Government to initiate the introduction of new information technology to the Health Service, it was decided to seek the views of local General Practitioners to use as a model in the planning of a future computerised discharge system for the area.

### METHODS

Postal questionnaires were sent out to all of the local General Practitioners in the local health care district. Each letter comprised an introductory page with an illustrated example of the format of the shorter discharge summary (see Figure 1) followed by a second page in which the Doctors were asked to indicate the importance they attached to various aspects of the discharge letter. This information was sought by means of a limited series of 7 questions related to the areas of speed of delivery, format, author, and content with a final "open" question inviting further comments. (See Figure 2).

Each question was responded to on a graduated scale of desirability, with a range of 5 possible options. These ranged from "essential" through "highly desirable", "desirable" and "not important" down to "undesirable".

The General Practitioners were drawn from a geographically defined health care district, served by 2 Consultants, each favouring a different discharge summary format. The General Practitioners were therefore divided into 3 groups according to the style of the summary they were familiar with receiving.

Thirty five Doctors received prioritised lists, 42 conventional longer summaries, while the remaining 22 General Practitioners received both formats.

**Figure 1**

BSG/VA/122478

1st January 1991

Dr Good
Best Surgery
Pinnacle Road
Barnstaple

Dear Dr Good,

Re: Amelia Smith, 14 03 05
1 South View Crescent, Barnstaple EX31 4JB

Admitted 21 12 90 — Discharged 01 01 91

| | |
|---|---|
| PROBLEMS | 1. Right Cerebral haemorrhage<br>2. Insulin dependent Diabetes Mellitus (1965)<br>3. Early dementia.<br>4. Arthritic husband. |
| PROGRESS | Admitted Hemiplegic. Good recovery to walk with Zimmer. Continent. Confusion less troublesome during OT home visit. |
| DRUGS AT DISCHARGE | Mixtard Insulin 35 u sc om<br>Fybogel one om |
| FUTURE PLANS | District Nurse and CPN asked to recommence visiting. Hospital discharge scheme help (Mon, Wed, Fri) Day Hospital (Tues, Thurs) |

Yours sincerely

Dr Gentle
Consultant Physician

Correspondence to: Dr. M.H. King, B.M. D.P.D., c/o The Westbank Practice, Church Street, Starcross, EXETER, EX6 8PZ

**Figure 2**

DISCHARGE SUMMARY QUESTIONNAIRE

Please indicate with a tick the relative importance you attach to the following:

1 = Essential
2 = Highly desirable
3 = Desirable
4 = NOT important
5 = Undesirable

(1) Speed of Discharge Summary — 1 2 3 4 5
Summary sent out: (a) Same day as Patient.
(b) Same week as Patient.
(c) Same month as Patient.

(2) Preferred Summary Format
(a) Emphasis on Brevity. Salient points only.
(b) Conventional comprehensive prose account.

(3) Details of ancillary 'non-acute' problems (including "Social" problems)

(5) Discharge details
(a) Medication.
(b) Methanical aids supplied.
(c) Rehabilitation arrangements.
(d) Social Services.
(e) Medical follow-up.
(f) Discharge destination.

(6) The flow of information
Details of information given to:
(a) The Patient.
(b) The next-of-Kin.

(7) Source of Summary
Summary written by:
(a) Consultant.
(b) SHO/REG (not checked by Consultant)
(c) SHO/REG (checked by Consultant)

IF YOU HAVE ANY FURTHER COMMENTS PLEASE RECORD THEM OVERLEAF.

### RESULTS

Analysis of the results revealed that all 99 General Practitioners returned their questionnaires. Of these, 88% favoured the shorter format, with 18% actually stating the "list" format to be essential. Only 27% of Doctors felt that conventional letters were desirable, with 20% of respondents indicating that "long" letters were undesirable. The trend towards shorter letters was repeated in all 3 groups, with no statistically significant differences between the groups.

The speed of arrival of summaries also proved to be an important area: 21% felt it essential that the letter should be sent out on the day of the patient discharge; whilst 83% felt this to be at least desirable. Three quarters of Doctors felt it desirable that the letter should be sent during the same week as discharge.

Discharge medication and destination both scored highly as essential information (90% and 57% respectively) whilst details of follow up arrangements, rehabilitation and Social Services also drew enthusiastic support, with 82%, 81% and 74% of General Practitioners scoring this as at least "highly desirable" information.

In contrast, those questions concerning details of ancillary "non-acute" problems and "normal" results of investigations provoked a more ambivalent response, with a small but significant number of Doctors indicating these areas as not important (11% and 17%).

The information given to the Patient and the next of kin, however, were much more highly rated in terms of importance, with 84% and 78% of those responding holding these as at least desirable areas to be covered in the summary.

General Practitioners were agreed that the author of the summary (Junior v Consultant) was a somewhat irrelevant issue, with up to a third of Doctors indicating this to be an unimportant point.

The Doctors' comments in response to our "open" question proved to be most revealing with a majority tending to concentrate heavily on the overall speed of the discharge summary process. Eleven Doctors indicated that the speed was the salient factor, while 9 positively endorsed the shorter "list" format.

### DISCUSSION

The response rate to this study clearly illustrates the importance that General Practitioners attach to the discharge summary process. In Hospital Medicine however the situation is less clear with a lower level of priority generally ascribed to the process, as evidenced by the fact that the dictating of summaries traditionally falls to the most junior member of the team.

This interesting dichotomy of priority and emphasis may lie at the heart of the malaise that bedevils the communication between Hospitals and General Practitioner.

Hospital Junior Doctors often with little or no first hand experience of General Practice may readily fail to fully appreciate the advantages of access to rapid and reliable discharge data. The use of longer prose style discharge letters can add an unnecessary extra burden to already hard pressed Junior Staff who frequently view the discharge letters as a rather peripheral duty.

The national Hospital preference for lengthy discharge letters was shown in a recent review which showed that 87% of Hospital Departments still favour a "dual" system, comprising an initial short note, followed by a longer letter. The remaining 13% of units have opted for using a single short summary[5]. Unfortunately this popular "two-tier" system is not without it's drawbacks; the most persistent of these being a tendency to "skimp" on the short note because a full letter is to follow[6].

Further studies have shown that 75% of departments still adhere to a "free-style" approach to letter dictation[5], although an increasing number are now beginning to develop discharge protocols in an attempt to standardise and improve the service offered to General Practitioners. Arguably there are clear advantages for Junior Hospital Doctors in adopting a departmental format. The structured framework helps to concentrate the thoughts of the author, ensuring that all the important basic information (both clinical and social) is included, helping to make the task considerably quicker, and less onerous. This in turn may have a significant "spin-off" in terms of modifying the notable lack of enthusiasm prominent amongst juniors in this area of their clinical responsibility.

Secretarial staff as well as Junior Doctors seem to respond positively to shorter summaries[3] being generally perceived as being quicker and easier to type. This is an important point as several studies have identified the typing stage as being one of the major "bottlenecks" in the system. A 1988 survey showed that in some units up to 5% of the total annual summary output might be unprocessed in the typing pool[7]. The re-motivation of secretarial staff is undoubtedly essential in any attempt to improve Hospital-General Practitioner communication.

It seems possible therefore, that appropriate modification of the discharge format may yield a significant upgrading of the service in a number of important areas. However, the responsibility for audit of hospital departmental policy inevitably lies with individual Consultants, who nationally have been slow in experimenting with progressive structured formats.

More recently the rate of progress has accelerated, partially due

*Continued on p. 55*

### THE CHANGING FACE OF BRISTOL MEDICINE

*Continued from p. 32.*

**Figure 1a** The relationship between NHS and University as it should be.

**Figure 1b** The relationship as it could develop.

### TOWARDS BETTER DISCHARGE SUMMARIES

*King*

West of England Medical Journal Volume 106)ii) June 1991

*Continued from p. 41*

to pressure from General Practitioners, but also stimulated by the gradual introduction of new information technology which is proving to be a potent catalyst for change in the system.

With a fundamental review of the discharge process in motion provoked by the information technology revolution, general practitioners may look to the future with some optimism as better quality discharge summaries seem a likely early outcome.

### ACKNOWLEDGEMENTS

I would like to express my thanks to Dr Stephen Barber for his valuable support in setting up this study, to Angela Vowden and Joand Homewood for typing the manuscript, and to all the General Practitioners who generously took part.

### REFERENCES

1. HARDING, J. (1987) Study of discharge communications from Hospital Doctors to an inner London General Practice. *J. R. Coll. Gen. Pract.*, **37**, 494–495.
2. MAGEEAN, R. J. (1986) Study of "Discharge communications" from hospital. *Br. Med. J.* **293**, 1283–1284.
3. EKELUND, P. and CASTLEDEN, C.M. (1989) Shorter and better c.scharge summaries. *Ger. Med.* **19**(12), 45–46.
4. PEARSON, S. *ET AL.* (1984) Letter. *Br. Med. J.* **289**, 189.
5. BLACK, D. (1990) How to do justice to discharge summaries. *Ger. Med.* **20**(9), 9.
6. HOWARD, D.J. (1986) Structured discharge letter in a department of Geriatric Medicine. *Health Trends*, **18**, 12–14.
7. PENNY, T.M. (1988) Delayed communication between hospitals and general practitioners: where does the problem lie? *Br. Med. J.* **297**, 28–29.

## Figures and Tables

**Figure 1 f1:**
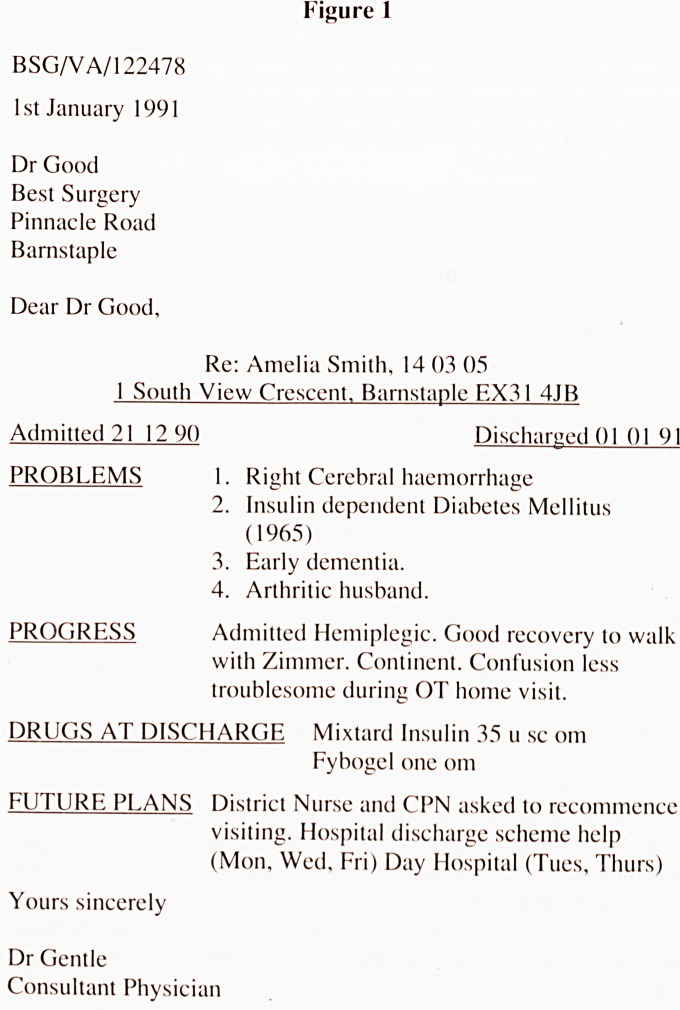


**Figure 2 f2:**